# Use of Hematopoietic Cell Transplant for Hematologic Cancers by Race, Ethnicity, and Age

**DOI:** 10.1001/jamanetworkopen.2024.33145

**Published:** 2024-09-18

**Authors:** Theresa Hahn, Megan M. Herr, Ruta Brazauskas, Jinalben Patel, Sikander Ailawadhi, Wael Saber, Nandita Khera

**Affiliations:** 1 Department of Cancer Prevention and Control, Roswell Park Comprehensive Cancer Center, Buffalo, New York; 2 Department of Medicine, Roswell Park Comprehensive Cancer Center, Buffalo, New York; 3 Division of Biostatistics, Medical College of Wisconsin, Milwaukee; 4 CIBMTR (Center for International Blood and Marrow Transplant Research), Medical College of Wisconsin, Milwaukee, Wisconsin; 5 Division of Hematology/Oncology, Mayo Clinic-Florida, Jacksonville; 6 Department of Hematology/Oncology, Mayo Clinic-Arizona, Phoenix

## Abstract

**Importance:**

Utilization of hematopoietic cell transplantation (HCT) for hematologic cancers previously demonstrated race, ethnicity, and age-based disparities.

**Objective:**

To evaluate utilization over time by race, ethnicity, and age to determine if disparities persist in light of recent significant increases in HCT volume.

**Design, Setting, and Participants:**

This US population-based retrospective cohort study includes patients who received transplants from January 2009 to December 2018. Data collection and cleaning occurred from February 2019 to November 2021, and data analysis occurred from January 2022 to October 2023. Method 1 restricted the analysis to Surveillance, Epidemiology and End Results (SEER) reporting areas for cases and transplants. Method 2 applied SEER age-, race-, and ethnicity-specific incidence rates to corresponding US census population and included all transplants reported to the Center for International Blood and Marrow Transplant Research. Race and ethnicity groups were hierarchically defined as Hispanic (any race), non-Hispanic White, non-Hispanic Black, and non-Hispanic Other (Asian and American Indian).

**Exposure:**

Receipt of HCT.

**Main Outcomes and Measures:**

Utilization rate of autologous or allogeneic HCT for patients with hematologic cancers by age, race, and ethnicity.

**Results:**

From 2009 to 2018, 136 280 HCTs were analyzed for 6 hematologic cancers comprising 16.7% pediatric/adolescent/young adults (0-39 years), 83.3% adults (40-84 years), 58% male, 10.3% Hispanic, 11.4% non-Hispanic Black, 3.8% non-Hispanic Other, and 74.5% non-Hispanic White patients, with 49 385 allogeneic and 86 895 autologous HCTs performed. HCT utilization increased over time for all disease, age, race, and ethnic groups. From 2017 to 2018, adult (40-84 years) allogeneic transplant utilization for acute myeloid leukemia and myelodysplastic syndrome (MDS) was similar for Hispanic and non-Hispanic White or Other patients but was lower for non-Hispanic Black patients (acute myeloid leukemia: 19% vs 13%; MDS: 9%-10% vs 5%). Similarly, autologous transplant utilization for lymphoma was similar for all race and ethnicity groups; however, autologous transplant for multiple myeloma was highest for non-Hispanic White patients and lower for all other groups (31% vs 26%-27%). In patients aged 0 to 39 years, utilization of allogeneic transplant for acute lymphoblastic leukemia was highest in Hispanic patients, followed by non-Hispanic White, Black, and Other races (acute lymphoblastic leukemia: 19%, 18%, 17%, and 16%, respectively).

**Conclusions and Relevance:**

In this cohort study of autologous and allogeneic transplant utilization for hematologic cancers, disparities persisted for non-Hispanic Black patients. Hispanic, non-Hispanic Other, and younger age groups had increased utilization over time that was on par with non-Hispanic White patients in the most recent cohort.

## Key Points

**Question:**

Has utilization of hematopoietic cell transplant (HCT) for hematologic cancers continued to demonstrate disparities over time by race, ethnicity, and age?

**Findings:**

In this cohort study of 136 280 patients with HCT, the rate of HCT for hematologic cancers in Hispanic and younger age groups increased over time and was on par with non-Hispanic White patients; however, non-Hispanic Black patients had lower utilization rates across all diseases.

**Meaning:**

These findings suggest progress has been made to narrow disparities in utilization of HCT for hematologic cancers in some groups; however, these findings highlight unmet needs for older adults and non-Hispanic Black patients of all ages.

## Teaser

This cohort study evaluates use of hematopoietic cell transplantation for hematologic cancers over time by race, ethnicity, and age to determine if disparities persist.

## Introduction

Hematopoietic cell transplantation (HCT) is an important treatment modality for hematologic cancers. Because of its medical complexity and high cost, disparities in utilization are well known.^1,2,3^ The Center for International Blood and Marrow Transplant Research (CIBMTR) is a national resource to assess HCT volume, but a denominator of patients with transplant-eligible diseases is required to examine utilization. Prior studies estimated the number of eligible cases by applying incidence rates from Surveillance Epidemiology and End Results (SEER) data to US census data to derive the denominator,^2,3,4^ with CIBMTR data providing the numerator of HCT volume.^3,4,5^ Alternatively, SEER Medicare data have been used to derive both the numerator and denominator, but this limits the study population to Medicare beneficiaries only.^6^A prior large CIBMTR study demonstrated significant increases in both volume and survival,^1,2,5,7^ but did not assess the impact of race, ethnicity, or age, nor did it determine if increases in volume or survival were equitable across these vulnerable subgroups.

We recently showed significant increases in volume for non-Hispanic White, non-Hispanic Black, and Hispanic patients and significant improvement in survival for all groups except non-Hispanic Black patients undergoing allogeneic HCT.^8^ Our current analysis sought to further expand our understanding of temporal changes in utilization of HCT by age, race, and ethnicity to identify where disparities persist. We used 2 methods of determining utilization to compare and contrast the methods and results.

## Methods

### Data Source for Numbers of HCTs

CIBMTR is a national resource which collects data pretransplant, 100 days, 6 months, and annually until year 6 posttransplant, and biannually thereafter on almost all US allogeneic HCTs and about 85% of autologous HCTs. Transplant centers obtain informed consent from each participant to use their data for research; written informed consent was a requirement for inclusion in this study. This study was reviewed and approved by the NMDP institutional review board. This report follows the Strengthening the Reporting of Observational Studies in Epidemiology (STROBE) reporting guidelines for cohort studies.

CIBMTR generated frequencies of first autologous and allogeneic HCTs from 2009 to 2018, divided into 5 two-year cohorts. Exclusion criteria were syngeneic HCTs; nonconsenting patients; embargoed center; or missing or unknown age, race, or ethnicity. For allogeneic HCT, the most common diseases were acute myeloid leukemia (AML), myelodysplastic syndrome (MDS), and acute lymphoblastic leukemia (ALL), and for autologous HCT, the most common diseases were non-Hodgkin lymphoma (NHL), multiple myeloma (MM), and Hodgkin lymphoma (HL). Analyses were performed for (1) pediatric, adolescent, and young adult (PED/AYA) patients aged 0 to 39 years, and (2) adults aged 40 to 84 years. Due to small numbers, results are not shown for allogeneic HCT for MDS and autologous HCT for MM for PED/AYA patients. Race and ethnicity were self-reported by the patient to the transplant center, which submitted those data to CIBMTR. Race and ethnicity groups were hierarchically defined as Hispanic (any race), non-Hispanic White (White), non-Hispanic Black (Black) and non-Hispanic all other races (Other race). The Hispanic group in the allogeneic HCT population was 94.7% White, 2.4% Black, and 2.9% Other race and in the autologous HCT group was 94.9%, 3.2%, and 1.5%, respectively. The Other race group with allogeneic HCT included 7% American Indian or Alaska Native, 87% Asian, and 6% Native Hawaiian or Pacific Islander, with corresponding proportions in autologous HCT of 11%, 84%, and 5%, respectively.

### Data Sources for Number of Cases

Two methods of population-based estimates for hematologic cancer cases were used, both leveraging SEER data. The SEER program provides information on cancer statistics, including incidence rates and incident cases.^9^ Eligible patients were aged 0 to 84 years and diagnosed with AML, MDS, ALL, NHL, HL, or MM during 2009 to 2018, as reported to 21 SEER registries (eTable 1 in Supplement 1).^10^ Hematologic cancer case counts were calculated from SEER*Stat using SEER-21 data. Case counts were tabulated by 5-year age-, race-, and ethnicity-specific groups (Black, Hispanic, White, and Other race).

### Method 1: SEER Analysis Using Actual Incident Cases for HCT Utilization in Hematologic Cancers

HCT volume was calculated for patients who had undergone HCT who resided in a SEER-21 area. Patient residential zip code reported to CIBMTR was matched to a county, then counties were matched to a SEER capture area. Patient zip code captured a transplant for a corresponding patient with hematologic cancer who was diagnosed in the same SEER area. Since zip code was required, an inflation factor was calculated to account for missing or incomplete zip code.^5^ The inflation factor was calculated as 1 / (No. of patients with available zip code / total No. HCT) for each 2-year cohort. The inflation factor was applied to the number of transplants in each cohort from 2009 to 2014 to account for a variable rate of missing zip codes over time (eTable 2 in Supplement 1). Transplants performed from 2015 to 2018 did not have an inflation factor applied because zip codes were available for most patients. The total hematologic cancer cases in SEER-21 by age, race, ethnicity, and year are presented in eTable 3 in Supplement 1.

### Method 2: US Census Analysis Using SEER Incidence Rates for Utilization of HCT for Hematologic Cancers

All HCTs reported to CIBMTR were included; zip code was not required, thus no inflation factor was applied. SEER*Stat using SEER-21 calculated the incidence of each hematologic cancer for each year from 2009 to 2018, each 5-year age cohort, and each race and ethnicity group (eTable 4 in Supplement 1). Incidence rates were then applied to the corresponding decennial or intercensal population estimates for the same age, race, and ethnicity category (eTable 5 in Supplement 1) to estimate the number of cases for each age group by race and ethnicity for each year (eTable 6 in Supplement 1). Hematologic cancer cases were then summed across age groups (0-39 and 40-84 years) and for each 2-year cohort to generate expected case numbers by race and ethnicity group over time (eTable 7 in Supplement 1).

### Statistical Analysis

For method 1, autologous and allogeneic HCT utilization rates were calculated by dividing the estimated number of HCTs performed within a SEER reporting area for each disease, age, race, and ethnicity group by the total number of associated hematologic cancer cases for the same age, race, and ethnicity group for the same SEER reporting area. For method 2, autologous and allogeneic HCT utilization rates were calculated by dividing the total number of reported HCTs performed in the US for each disease, age, race, and ethnicity group by the estimated number of associated hematologic cancer cases in the US for the same age, race, and ethnicity group. To describe changes over time in hematologic cancer case counts, HCT volume, and HCT utilization rates (eTable 3, eTable 8, and eTable 9 in Supplement 1), the terminal anchor cohorts (2009-2010 and 2017-2018) were compared by dividing the count, volume, and rate from 2017 to 2018 by that from 2009 to 2010 and multiplying by 100 to yield the percentage change over time. A change greater than 120% defined increase and was described as increased by 20%, less than 90% defined decrease and was described as decreased by 10%, and 90% to 119% was described as stable over time (100% equals no change). Statistical significance was not calculated and measures of variance are not applicable in this descriptive population-based study. Data were analyzed from January 2022 to October 2023.

## Results

### Total US Population

From 2009 to 2018, 136 280 HCTs were analyzed for 6 hematologic cancers comprising 16.7% pediatric/adolescent/young adults (0-39 years), 83.3% adults (40-84 years), 58% male, 10.3% Hispanic, 11.4% non-Hispanic Black, 3.8% non-Hispanic Other, and 74.5% non-Hispanic White patients, with 49 385 allogeneic and 86 895 autologous HCTs performed. Per US census data, from 2009 to 2018, the White population was stable at about 197 000 000 people (increased by 0.13%, net >260 000 people) while the Hispanic population increased by 20.9% (net >10 000 000 people), the Black population increased by 8.5% (net >3 200 000 people) and the Other race population increased by 26% (net >4 400 000 people) (eTable 5 in Supplement 1). In 2009, White persons made up 64.3%, Hispanic 16.1%, Black 12.3%, and Other race 5.6% of the US population compared with 60.5%, 18.2%, 12.5%, and 6.6%, respectively, in 2018.

In 2009, people aged 0 to 19 years were the largest proportion among the Hispanic (37.7%) and Black (31.6%) populations compared with Other race (26.2%) and White (23.1%) populations (eTable 5 in Supplement 1). These proportions decreased for all groups by 2018 (34.6%, 27.6%, 23.1%, and 21.1%, respectively). In 2009, people aged 65 to 84 years were the largest proportion in White (13.8%), followed by Other race (8.2%), Black (7.8%), and Hispanic (4.9%) groups. These proportions increased for all groups by 2018 (17.7%, 11.1%, 10.5%, and 6.6%, respectively). These demographic changes with lower proportions in the youngest ages and higher proportions in the oldest ages demonstrate an aging US population during this time.

### Method 1: SEER-Restricted Analysis for HCT Utilization for Hematologic Cancers

In PED/AYA patients (aged 0-39 years), hematologic cancer cases reported to SEER were stable over time for all diseases in Hispanic patients, increased for AML and ALL in Other race patients, and decreased by more than 10% in Black patients for HL and White patients for AML and NHL (eTable 3 in Supplement 1). The allogeneic HCT volume and utilization rate for ALL increased across all race and ethnicity groups, whereas for AML, allogeneic HCT volume decreased by more than 20% for Other race and White patients while the utilization rate decreased for Black and Other race patients. Autologous HCT volume and rates at least tripled for HL in Hispanic and Other race patients, increased more than 30% in volume and rate among Black patients with HL, increased more than 20% in volume and rate among Hispanic patients with NHL, and decreased more than 15% in volume among Black patients with NHL and in volume and rate among Other race patients with NHL.

In the most recent cohort from 2017 to 2018, Black PED/AYA patients had lower rates of HCT than White patients for AML (30% vs. 46%), ALL (15% vs 16%), and NHL (4% vs 5%) but higher rates for HL (14% vs 8%). Hispanic patients had lower rates of HCT than White patients for AML (38% vs 46%), similar rates for NHL (5% vs 5%), and higher rates for ALL (19% vs 16%) and HL (12% vs 8%). Other race patients had lower rates than White patients for all 4 indications (eTable 3 in Supplement 1).

In adults aged 40 to 84 years, case counts reported to SEER for every hematologic cancer were stable in White and increased in Other race patients, with Black and Hispanic patients experiencing increases in at least half the diseases (eTable 3 in Supplement 1). Corresponding increases over time were also seen in the allogeneic HCT volume and utilization rates for all diseases and race and ethnicity groups, except that the HCT rate for AML in Other race patients was stable. Autologous HCT volume and rates increased for most disease and race and ethnicity groups with a notable decrease of about half the HCT volume and utilization rate for HL in Black patients. (eTable 3 in Supplement 1).

In the most recent cohort from 2017 to 2018, Black adult patients had lower rates of HCT than White patients for all 6 diseases (AML: 11% vs 19%; MDS: 4% vs 10%; ALL: 17% vs 32%; MM: 20% vs 25%; HL: 4% vs 6%; and NHL: 3% vs 4%). Hispanic patients had lower rates of HCT than White patients for AML (17% vs 19%) and MM (11% vs 25%), similar rates for NHL (4%) and HL (6%), and higher rates for ALL (33% vs 32%). Other race patients had lower rates than White patients for all 6 HCT indications (eTable 3 in Supplement 1).

### Method 2: US Population Analysis for HCT Utilization for Hematologic Cancers

In the PED/AYA group, hematologic cancer volume in Black patients was stable for ALL and NHL, increased for AML, and decreased for HL. HCT volume for all 4 diseases increased by more than 25% while allogeneic HCT utilization rates were stable and autologous HCT rates increased by more than 30% for HL and NHL (eTable 8 in Supplement 1). In Hispanic patients, the case volume of all 4 diseases was stable, while the HCT volume increased for all diseases by more than 30%, and allogeneic HCT for AML and ALL and autologous HCT for HL increased by more than 25%. In Other race patients, case counts of all 4 diseases increased, with corresponding increases in HCT volume for all except allogeneic HCT for AML, which remained stable, resulting in a lower utilization rate in AML but stability in the other 3 indications. In White patients, case counts remained stable or decreased for NHL, with corresponding HCT volume remaining stable or decreased for NHL and stable utilization rates over time (eTable 8 in Supplement 1).

In the most recent cohort from 2017 to 2018, Black PED/AYA patients had lower HCT rates than White patients for AML (37% vs 53%), ALL (17 vs 18%), and NHL (5% vs 6%) but higher rates for HL (15% vs 11%). Hispanic patients had lower HCT rates than White patients for AML (41% vs 53%) and NHL (5% vs 6%) but higher rates for ALL (19% vs 18%) and HL (14% vs 11%). Other race patients had lower rates than White patients for 3 of the 4 indications (eTable 8 in Supplement 1).

In Black adults, case counts for AML, ALL, and MM increased while MDS, HL, and NHL remained stable; HCT volume and rates increased for all diseases except HL, which remained stable. In Hispanic adults, case counts for AML, ALL, MM, and NHL increased while MDS and HL remained stable; HCT volume and utilization rates increased 38% or more for all diseases, with the HCT rate for MDS and HL at least doubling. In Other race adults, case counts and HCT volume increased for all 6 diseases; however, utilization rates increased for 4 of 6 diseases, with allogeneic HCT rates for AML and ALL remaining stable due to proportional increases in both case counts and HCT volume. In White adults, case counts were stable for all diseases except ALL, which increased; HCT volume increased for all diseases except HL and NHL, which remained stable; HCT utilization rates increased for MDS, ALL, and MM; and rates remained stable for AML, HL, and NHL (eTable 9 in Supplement 1).

**Figure 1.**
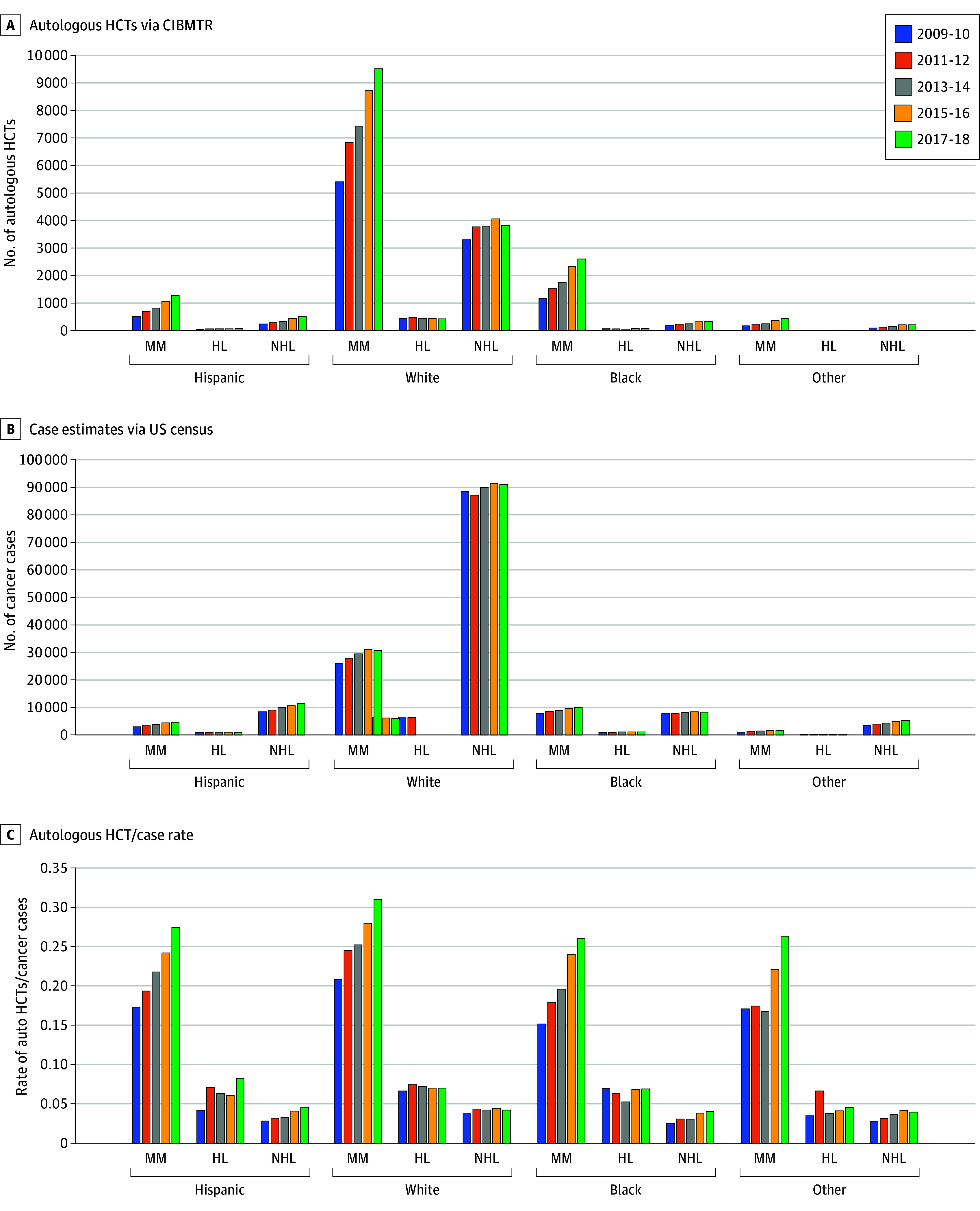
Autologous Hematopoietic Cell Transplantation (HCT), Hematologic Cancer Case Volume, and Autologous HCT Rate in Adults by Disease, Race and Ethnicity, and Year In adult patients (40-84 years), the volume of autologous HCT cases reported to CIBMTR was highest in White compared with all other race and ethnic groups (A) and was similar to hematologic cancer cases (B). C, Rates of autologous HCTs/hematologic cancer cases increased over time for most groups but still demonstrated disparities for minority racial and ethnic groups with MM or HL. Other race includes American Indian or Alaska Native, Asian, and Native Hawaiian or Pacific Islander. CIBMTR indicates Center for International Blood and Marrow Transplant Research; HL, Hodgkin lymphoma; MM, multiple myeloma; NHL, non-Hodgkin lymphoma.

**Figure 2.**
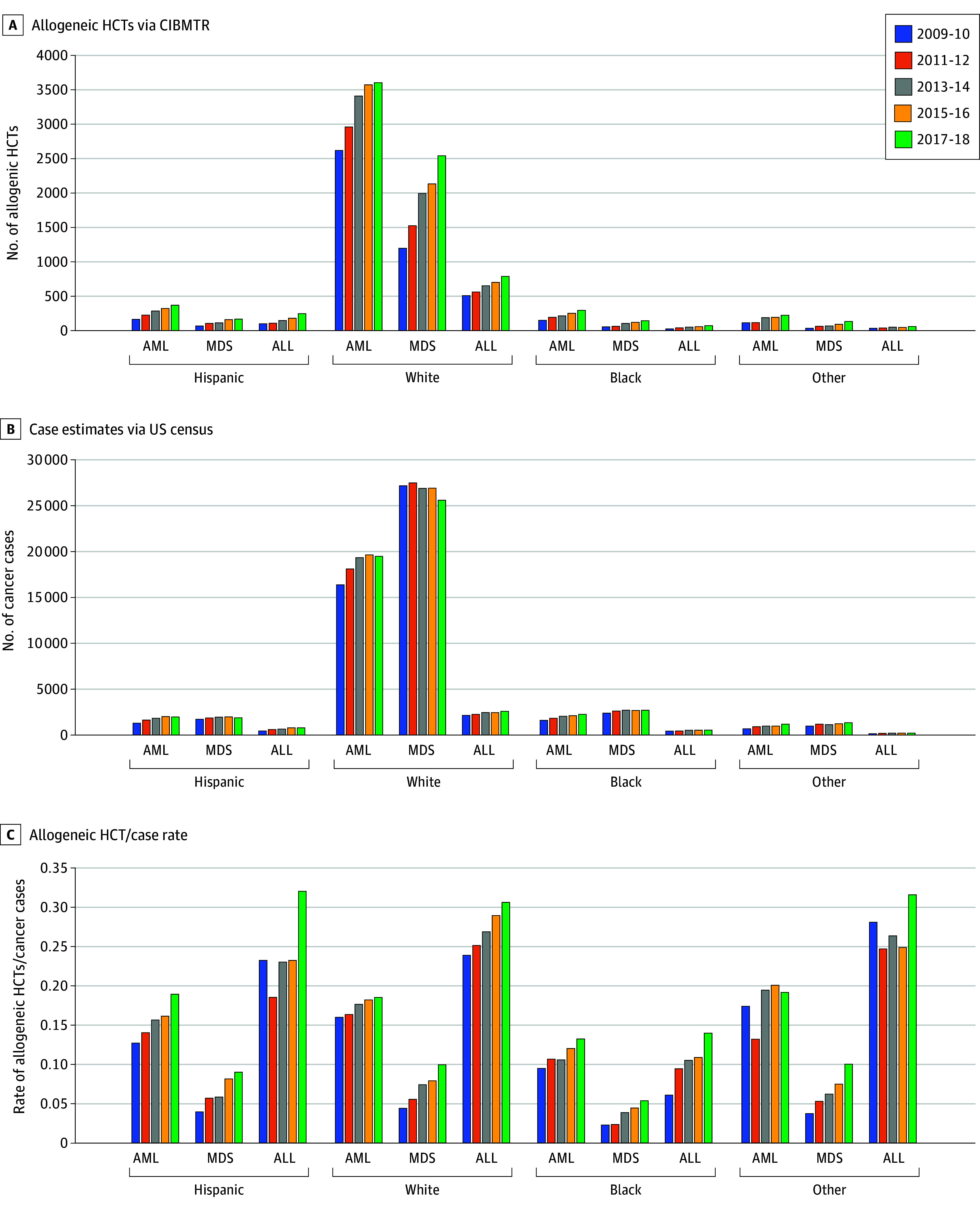
Allogeneic Hematopoietic Cell Transplantation (HCT), Hematologic Cancer Case Volume, and Allogeneic HCT Rate in Adults by Disease, Race and Ethnicity, and Year In adult patients (40-84 years), the volume of allogeneic HCT cases reported to CIBMTR was highest in White compared with all other racial and ethnic groups (A) and was similar to hematologic cancer cases (B). C, Rates of allogeneic HCTs or hematologic cancer cases increased over time for all groups but continued to demonstrate pronounced disparities for Black patients with AML, MDS, and ALL. Other race includes American Indian or Alaska Native, Asian, and Native Hawaiian or Pacific Islander. ALL indicates acute lymphoblastic leukemia; AML, acute myeloid leukemia; CIBMTR, Center for International Blood and Marrow Transplant Research; MDS, myelodysplastic syndrome.

Autologous HCT rates for MM increased for all race and ethnicity groups while rates for NHL increased for Black patients; HL and NHL increased for Hispanic and Other race patients and were stable for White patients (Figure 1). Utilization of autologous HCT for MM is 19% higher in White vs Black patients. Allogeneic HCT rates for AML, MDS, and ALL increased over time for all race and ethnicity groups except Other race rates of AML and ALL and White rates of AML, which were stable (Figure 2). From 2017 to 2018, Black adult patients had lower HCT rates than White patients for AML, ALL, MDS, and MM, with similar rates for HL and NHL. Hispanic patients had lower HCT rates than White patients for MDS and MM, similar rates for AML and higher rates for ALL, NHL, and HL. Other race patients had lower HCT rates than White patients for MM and HL, but similar or higher rates for AML, MDS, NHL, and ALL (eTable 9 in Supplement 1, Figure 1 and Figure 2).

### Comparison of 2 Methods Using SEER-Restricted and US Population for Utilization of HCT for Hematologic Cancer

SEER purposefully captures a higher proportion of cancer cases in minoritized race and ethnicity groups, and a lower proportion in White persons.^11^ This is demonstrated when comparing hematologic cancer cases in eTable 3 (SEER) with eTable 8 (PED/AYA US Census) and eTable 9 (adult US Census) in Supplement 1. From 2017 to 2018 for the PED/AYA group, SEER captured 33% to 34% of hematologic cancer cases in White patients, which was similar to Black patients (35%-36%) but was much higher for Hispanic patients (42%-45%) and Other race patients (59%-61%). These proportions did not change over time for White patients (33%-36%) or Black patients (35%-36%) but decreased for Hispanic patients (46%-48%) and Other race patients (66%-73%). From 2017 to 2018 in adults, SEER captured a lower proportion (24%-30%) of hematologic cancer cases in White patients than Black patients (29%-35%), Hispanic patients (37%-45%), and Other race patients (51%-62%). These proportions did not change over time for White patients (24%-30%), Black patients (30%-35%), or Hispanic patients (38%-46%) but decreased for Other race patients (57%-65%). Thus, SEER captures a variable and low proportion of the total hematologic cancer cases in the US by race and ethnicity.

**Figure 3.**
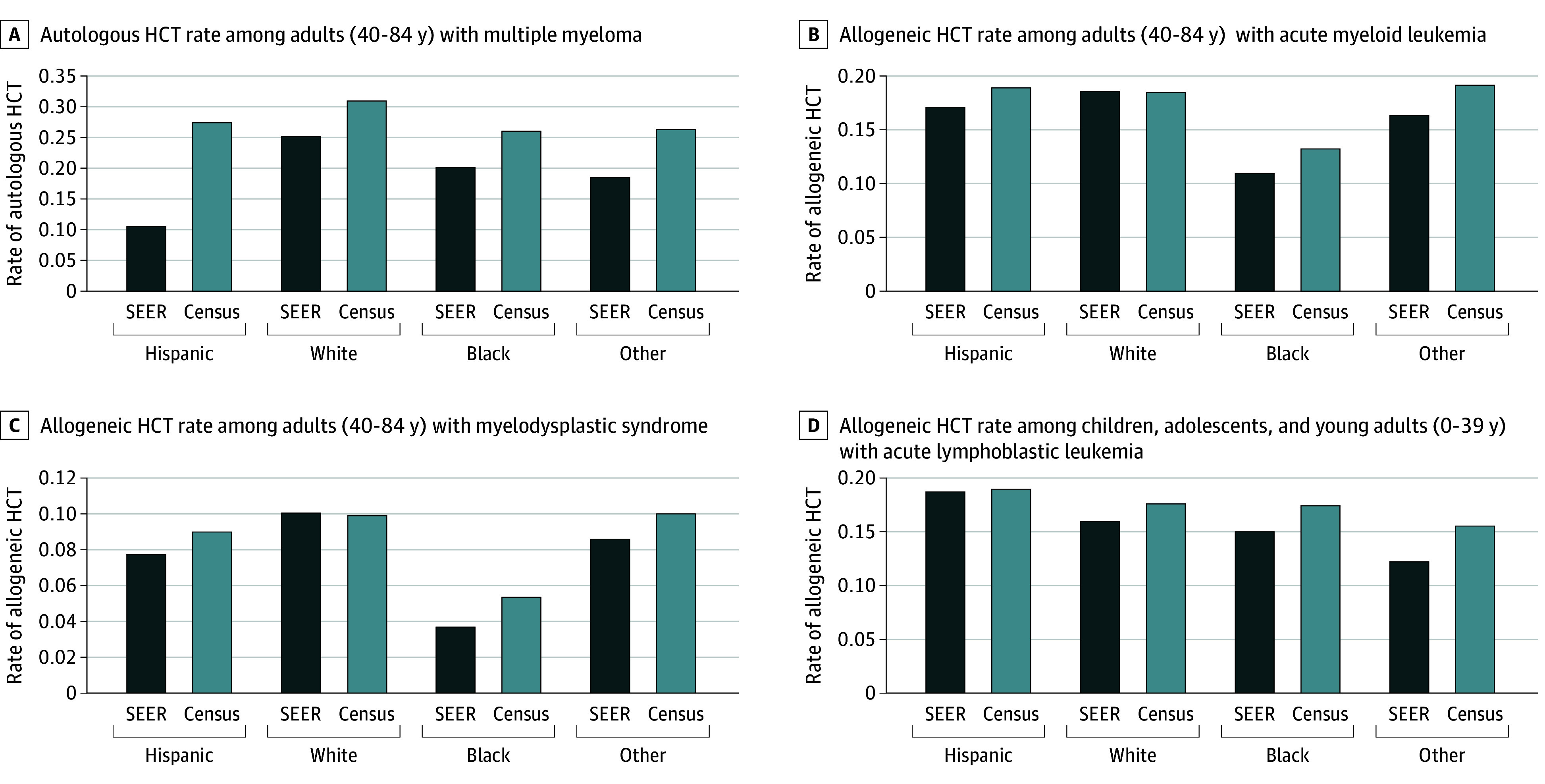
Surveillance, Epidemiology and End Results (SEER)–Restricted vs US Population Hematopoietic Cell Transplantation (HCT) Rates by Race and Ethnicity for Selected Diseases in Adult, Pediatric, and Young Adult Patients Treated 2017 to 2018 Comparing the SEER-restricted analysis with the US-based population method, the autologous HCT rate in adults with multiple myeloma was underestimated at varying degrees for all groups (A). The allogeneic HCT rate was underestimated in all minoritized groups but not in White patients for adults with acute myeloid leukemia (B) and not in adults with myelodysplastic syndrome (C). D, The allogeneic HCT rate was underestimated in all groups except Hispanic for pediatric and young adult patients with acute lymphoblastic leukemia. Other race includes American Indian or Alaska Native, Asian, and Native Hawaiian or Pacific Islander.

Allogeneic and autologous HCT utilization rates for each hematologic cancer, age, race and ethnicity group were variably over- and underestimated by method 1 by race, ethnicity and disease. For example, in the 2017 to 2018 cohort, method 1 underestimated the autologous HCT rate vs method 2 in adults with MM for Black (20% vs 26%), Hispanic (11% vs 27%), Other (19% vs 26%), and White (25% vs 31%) patients. Similarly for allogeneic HCT utilization rates, method 1 underestimates the rate of AML and MDS in adults across all minoritized groups (Black AML: 11% vs 13%; MDS: 4% vs 5%; Hispanic AML: 17% vs 19%; MDS: 8% vs 9%; Other AML: 16% vs 19%; MDS: 9% vs 1%) and more accurately estimates rates in White people (AML: 19% vs 19%; MDS: 10% vs 10%) (Figure 3; eTable 10 in Supplement 1). The allogeneic HCT utilization rate in PED/AYA for ALL was underestimated by method 1 in most race and ethnicity groups over time with a few anomalies in earlier time periods.

With respect to detection of disparities, both methods detected disparities between Black vs White PED/AYA patients, but method 1 missed a slight disparity in HCT for NHL in Hispanic and Other race patients that was detected with method 2 (eTable 10 in Supplement 1). In adult patients, method 2 detected disparities between Black vs White patients in 4 of 6 diseases, but method 1 underreported the HCT rate for HL and NHL in Black but not White patients, thus creating a disparity for HL and NHL only with method 1. Similarly, for Hispanic adult patients, method 1 demonstrated a lower HCT rate in Hispanic vs White patients with AML (0.17 vs 0.19), creating a disparity for this HCT indication that had equivalent rates using method 2 (0.19 vs 0.19). A similar pattern was seen in Other race adult patients, which demonstrated disparities for MM and HL with method 2 but had disparities for all 6 diseases with method 1.

## Discussion

SEER is an incredibly valuable national resource to track incidence and mortality rates and estimated cancer burden over time for the US. As with all data sources, understanding the strengths and limitations is key to applying this information to specific conditions that were not originally intended for the data source. Our application of estimating autologous and allogeneic HCT utilization for selected hematologic cancers in SEER-21 areas demonstrates variable under- and overestimation of utilization compared with method 2 using the whole US population.

Our study demonstrates the error of estimating racial and ethnic disparities when only focusing on the numerator (ie, the number of transplants). For example, MM has approximately twice the incidence rate in Black vs White people; however, MM cases are 3 times higher in White vs Black people due to there being approximately 5 times more White vs Black people in the US. Autologous HCT utilization for MM is 19% higher in White vs Black patients, suggesting a disparity in the most recent cohort (Figures 1C and 2C), but that disparity is not on the same scale as the incidence rate, MM case, or HCT volume.

A prior CIBMTR study used a similar method applying SEER-18 incidence rates to the US population in each major race and ethnicity subgroup to estimate utilization of autologous HCT for MM.^4^ However, it did not account for differences in age distribution by race and ethnicity. We showed the Hispanic population is much younger than the White population, with MM having a much higher incidence in older ages. Thus, our current application of incidence rates including 5-year age cohorts yielded a more precise and age-adjusted estimation of the total hematologic cancer cases to use as the denominator.

It is interesting to note the presence of disparities based on age and disease in our study. For example, allogeneic HCT rates for AML in PED/AYA are higher than for adults. While utilization has increased for both age groups across all racial and ethnic groups over time, they remain lower for Black patients in both age groups, similar to a recent report.^12^

Our study also highlights the unmet need for allogeneic HCT in diseases that affect older people, such as MDS.^13,14^ Recently, allogeneic HCT provides better outcomes than nontransplant treatment for high-risk patients with MDS,^14,15^ which accounts for 25% to 30% of all patients with MDS.^16,17^ We assume that HCT utilization for MDS has increased since these publications; however, further work is necessary to improve upon the 5% to 10% utilization rate from 2017 to 2018.^13,14^ Barriers and opportunities for expansion of allogeneic HCT for MDS have been described that can be extrapolated to other diseases and/or transplant indications with gaps in utilization.^18^

While no treatment should have 100% utilization, minoritized groups should have a similar utilization rate as White patients if equal access to HCT is available to all groups. Our quantitative analysis of these disparities provides evidence for further examination of the association of contextual factors with differential HCT utilization. Structural and systemic racism, defined as “societal systems and structures that expose people of color to health-harming conditions and that impose and sustain barriers to opportunities that promote good health and well-being,”^19^ is well known to be a mediator of disparities in health care.^13,20^

### Limitations

Our study has limitations. It demonstrates the outcomes of limiting the utilization analysis to SEER-reporting areas on the estimation of HCT utilization by race and ethnicity. Due to the geographic and demographic differences in SEER-reporting areas and location of centers that perform HCTs, the resulting study subpopulation may be biased and not representative of the full US population. Hence, utilization estimates obtained by the SEER-specific method may not be generalizable to the US population.

An additional limitation with both methods is *International Classification of Diseases for Oncology, 3rd Edition* codes to define hematologic cancer cases, which may lead to an underestimation of the true HCT utilization. For example, plasma cell myeloma (code 9732/3) includes MM, plasma cell leukemia, monoclonal gammopathy of uncertain significance, and smoldering MM, however only MM is treated with autologous HCT. Therefore, the estimated and actual number of cases of MM in SEER is diluted by other plasma cell disorders. Similarly, patients with AML with favorable risk disease in first complete remission may not need an allogeneic HCT but are included in the denominator, thus underestimating HCT utilization in high-risk AML. Nevertheless, these underestimations affect all time periods and race and ethnicity groups the same, hence our results still add value to the field. This limitation highlights the need to identify methods to determine more accurate denominators for disease indications for transplant per the current guidelines.^21^

While we were able to examine different age groups in SEER data for cases, we did not include information about other potential confounders such as sex, socioeconomic status, or language proficiency, which may also impact referral patterns and eventually utilization. Finally, attribution of race and ethnicity in the CIBMTR database is provided by patient self-report via the transplant center. While patient self-report is usually considered the gold standard, it does not uniformly correlate with genetic ancestry.

## Conclusions

In this study, HCT volume increased over time in all age, race, and ethnicity groups for most hematologic cancers. With variable changes in volume of hematologic cancer cases, there were improvements in HCT utilization that have narrowed disparities between some race and ethnicity groups. Utilization of HCT as a treatment modality was lower for Black patients with MDS, MM, ALL, and AML. More work is needed to understand and address the contextual factors to improve HCT utilization based on the latest scientific evidence and ensure equal access for disadvantaged populations.

## Notes

Supplement 1. **eTable 1.** *ICD-O-3* Morphology Codes Used to Define Each Hematologic Cancer Case in SEER-21 Areas, Grouped by World Health Organization Classification **eTable 2.** Inflation Factor Calculation for Method 1 Analysis **eTable 3.** Method 1—SEER-Restricted Analysis for Ped/AYA and Adult Patients **eTable 4.** Incidence Rates per 100 000 Population Calculated Using SEER*STAT for Each Hematologic Cancer by Age, Race, Ethnicity, and Year **eTable 5. **US Census Estimates of Total Populations by Age, Race, Ethnicity, and Year **eTable 6.** Hematologic Cancer Cases by 5-Year Age Groups, Race, Ethnicity, and Year of Diagnosis, Estimated Using US Census Data and SEER Incidence Rates **eTable 7. **Hematologic Cancer Cases by Major Age Groups, Race, Ethnicity, and Year of Diagnosis, Estimated Using US Census Data and SEER Incidence Rates **eTable 8.** Frequency of Transplants and Hematologic Cancer Cases, and Rate of Allogeneic and Autologous HCTs/Case Rates for Pediatric/AYA Patients Aged 0 to 39 Years Using US Census Data **eTable 9. **Frequency of Transplants and Hematologic Cancer Cases, and Rate of Allogeneic and Autologous HCTs/Case Rates for Adults Aged 40-84 Years Using US Census Data **eTable 10.** Comparison of Rates of Autologous and Allogeneic HCT by Disease, Race, Ethnicity, Year, and Age, Determined by Method 1 vs Method 2

Supplement 2.
Data Sharing Statement
