# Supplementary material for: Use of Hematopoietic Cell Transplant for Hematologic Cancers by Race, Ethnicity, and Age
Source: JAMA Netw Open. 2024 Sep 18;7(9):e2433145. doi: 10.1001/jamanetworkopen.2024.33145 (PMC11411389; doi:10.1001/jamanetworkopen.2024.33145)
Supplement: Supplement 2. — Data Sharing Statement [file jamanetwopen-e2433145-s002.pdf]

Data Sharing Statement

Hahn. Use of Hematopoietic Cell Transplant for Hematologic Malignant Tumors by Race, Ethnicity, and Age. *JAMA Netw Open*. Published September 18, 2024. doi:10.1001/jamanetworkopen.2024.33145

Data
Data available: Yes
Data types: Deidentified participant data
How to access data: the data and statistical calculations are provided in the supplemental tables to be publicly available when the manuscript is published
When available: With publication

Supporting Documents
Document types: Statistical/analytic code
How to access documents: the data and statistical calculations are provided in the supplemental tables to be publicly available when the manuscript is published
When available: With publication

Additional Information
Who can access the data: anyone who would like to download the data
Types of analyses: for any purpose
Mechanisms of data availability: no approval or request is needed
Any additional restrictions: no restrictions
